# miRNA-1283 Regulates the PERK/ATF4 Pathway in Vascular Injury by Targeting ATF4

**DOI:** 10.1371/journal.pone.0159171

**Published:** 2016-08-18

**Authors:** Ling He, Jing Yuan, Qingyun Xu, Ruixue Chen, Liguo Chen, Meixia Fang

**Affiliations:** 1 Department of Chinese Medicine, Medical College of Jinan University, Guangzhou, 510632, Guangdong Province, PR China; 2 Institute of Laboratory Animals, Jinan University, Guangzhou, 510632, Guangdong Province, PR China; University of Kansas School of Medicine, UNITED STATES

## Abstract

**Background:**

In our previous study, we found significant differences in the mRNA and microRNA (miRNA) levels among hypertensive patients with different degrees of vascular endothelial cells damage. These differences were closely associated with endoplasmic reticulum stress (ERS)-related proteins. Moreover, compared to the control group, the expression of transcription factor activating factor 4 (ATF4) was also found to be significantly different in the hypertensive patients with different degrees of vascular endothelial cells damage groups. These results were confirmed using gene prediction software, which showed synergistic effects between ATF4 and miR-1283. ATF4 is a key molecule in ERS. Three ERS pathways exist:protein kinase RNA-like ER kinase (PERK), activating transcription factor 6 (ATF6) and inositol-requiring enzyme-1 (IRE-1)-induced apoptosis. The PERK pathway is the most important and also includes the phosphorylation of eukaryotic translation initiation factor 2α (eIF2α) and ATF4. In this report, we studied the regulatory effects of miR-1283 and ATF4 on the PERK-eIF2α-ATF4 signaling pathway using human umbilical vein endothelial cells (HUVECs) and mice.

**Methodology/Principal Findings:**

We verified the relationship between miR-1283 and ATF4 using a luciferase activity assay and observed the regulatory effects of miR-1283 and ATF4 on the PERK-eIF2α-ATF4 signaling pathway in vivo and in vitro.

**Conclusions/Significance:**

ATF4 is a target gene of miR-1283, which regulates the PERK-eIF2α-ATF4 signaling pathway by inhibiting ATF4, and it plays a critical role in inducing injury in HUVECs and mouse heart tissue.

## Introduction

**Fig 1:**
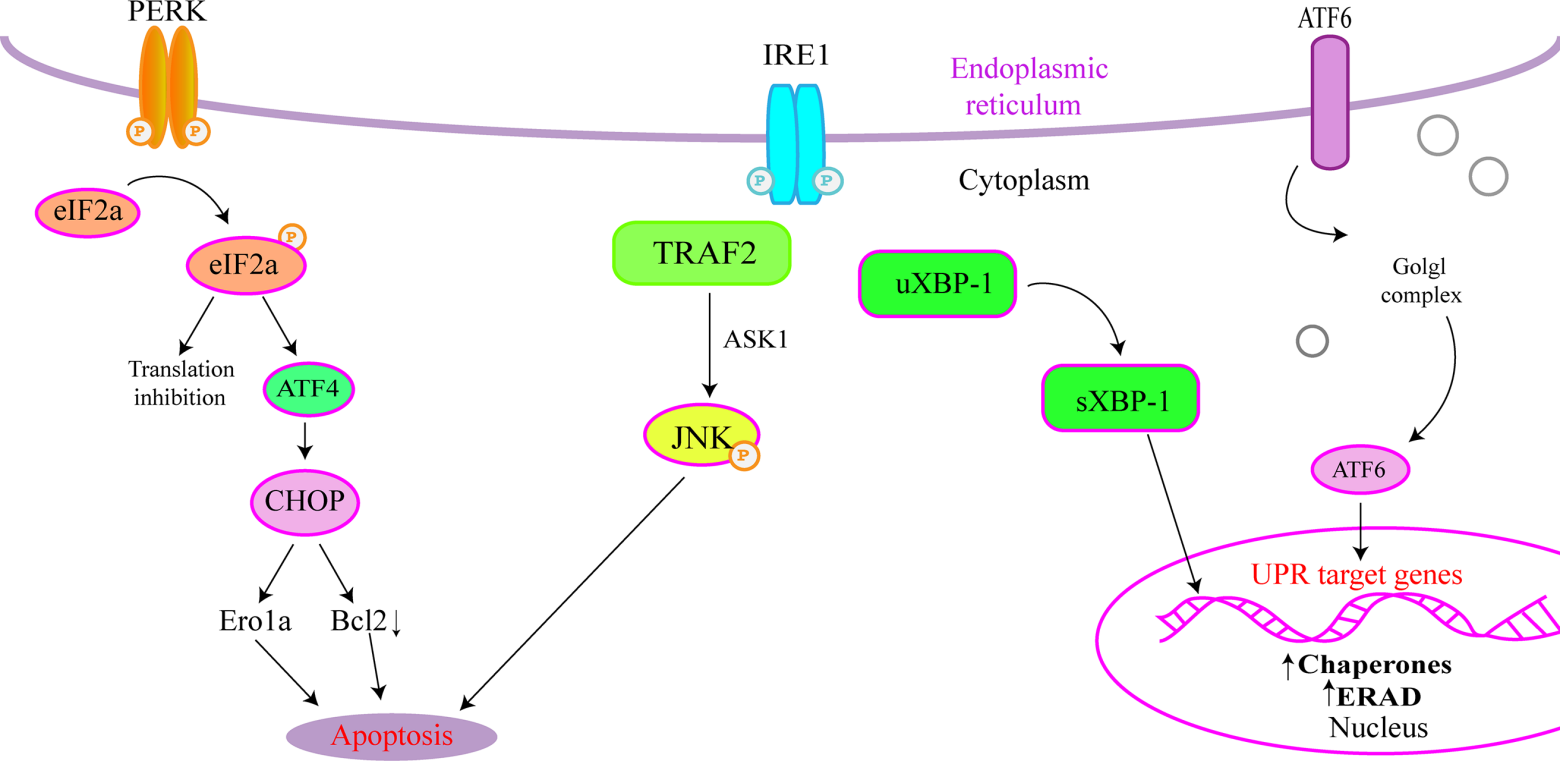
Endoplasmic reticulum stress pathway.

miRNAs are short, non-coding RNA molecules that are approximately 21–24 nucleotide (nt) in length. They inhibit effective mRNA translation of the target genes via imperfect base pairing with the 3'-untranslated region (3'UTR) of the target mRNA [1]. Changes in vascular endothelial cell (VEC) function have recently become a topic of interest. Numerous studies have shown that miRNAs play an important role in hypertension related endothelial dysfunction and the ability to decrease blood vessel diameter. VECs have a highly developed endoplasmic reticulum (ER) and are very sensitive to endoplasmic reticulum stress (ERS). Studies have shown that ERS is involved in the pathophysiological processes of vascular injury diseases, and intervention during ERS may be a new strategy for the treatment of cardiovascular diseases [2]. Ding has stated that apoptosis of VECs is merely the initial stage of atherosclerosis, and the ERS signaling pathway plays an important role in the regulation of VEC dysfunction [3]. Cell apoptosis can be induced by three proteins: protein kinase RNA-like ER kinase (PERK), activating transcription factor 6 (ATF6), and inositol-requiring enzyme-1 (IRE-1). The PERK pathway also involves the phosphorylation of eukaryotic translation initiation factor 2α (eIF2α) and ATF4, which is necessary for activation of C/EBP homologous protein (CHOP) (Fig 1).

In previous studies performed by our laboratory, we have shown that the pathogenesis of damaged vascular endothelial cells is related to ERS and involves the ATF4, ATF3, DDIT3, TRIB3, CEBPB and JUN genes. We found that ATF4 expression is higher in damaged vascular endothelial cells than in normal cells. However, miR-1283 levels are lower in damaged cells than in normal cells [4]. Target gene prediction using RNAhybrid (http://bibiserv.techfak.uni-bielefeld.de/rnahybrid/) and miRanda (http://www.microrna.org/microrna/home.do) showed that there are synergistic effects between ATF4 and miR-1283 [5]. Therefore, in this study, we used HUVECs and mice to investigate the role of miR-1283 and ATF4 in vascular endothelium injury.

## Materials and Methods

### Reagents

HUVECs were purchased from ATCC^®^ (CRL-1730, USA). Dulbecco's modified Eagle's medium (DMEM), fetal bovine serum (FBS) and phosphate belanced solution (PBS) were purchased from Gibco (Gibco, Grand Island, NY, USA). TRIzol and Lipofectamine^TM^ 2000 were purchased from Invitrogen (Invitrogen, Carlsbad, CA, USA). The miR-1283 mimic, miR-1283 antagomir and miRNA antagomir negative control were purchased from Guangzhou RiboBio Co., Ltd. (Guangzhou, China). The pmirGLO Dual-Luciferase miRNA target expression vector and the Griess reagent system were purchased from Promega (Promega, Madison, CA, USA). The quantitative real-time polymerase chain reaction (qRT-PCR) reagents were purchased from TaKaRa (TaKaRa, Japan). The GRP78, PERK, ATF4, CHOP, BCL2, BAX, GAPDH, and β-actin primers were synthesized by the Shanghai Generay Biotech Co., Ltd. (Guangzhou, China). The endothelin (ET), von Willebrand factor (vWF), thrombomodulin (TM), and EPCR enzyme-linked immunosorbent assay (ELISA) kits were purchased from RD Co., Ltd. (RD, Olean, NY, USA), and 4-PBA (4-sodium phenylbutyrate) was purchased from Sigma (Sigma, St. Louis, MO, USA). The PERK, eIF2α, p-eIF2α, ATF4, CHOP, BCL2, and BAX monoclonal antibodies were purchased from the CST Biotechnology Company (Cell Signaling Technology, Boston, MA, USA). The terminal deoxynucleotidyl transferase dUTP nick end labeling (TUNEL) apoptosis kit was purchased from Roche (Roche, Germany).

### CRL-1730 cell culture

After the cells reached confluence in the tissue culture flasks, we discarded the supernatant, washed the cells 3 times with PBS, digested the cells for 1 minute with 0.25% trypsin, and observed the cells under an inverted microscope. When most of the cells close to the wall had become rounded, we rapidly added 1 ml of DMEM containing 10% FBS to terminate the digestion reaction. Then, we gently collected these cells using a pipette, and centrifuged them at 1000 rpm at 4°C for 5 minutes. Next, we added 3 ml DMEM containing 10% FBS to produce a single cell suspension with a cell propagation ratio of 1:3. The cells were cultured at 37°C with 5% CO_2_ in a humidified incubator.

### Targeting the relationship between miR-1283 and ATF4

Primers targeting the sequences of ATF4, its 3'UTR and miR-1283 were designed using the NCBI database, and the products were used to obtain the 3'UTR of the target gene (Table 1). The relative activities of ATF4 and miR-1283 were determined with a dual-luciferase reporter assay.

**Table 1:** ATF4 primer information table.

Primer name	Sequence(3'-5')	Annealing uniformity(°C)	Product length (bp)
**ATF4-UTR**	F:TAGCTAGCTAGCTGCCCGTCCCAAACCTTAC	60	533
	R:CTCTAGAGCTCCGAGCAGGGATGAGCAAAT		
**ATF4-UTR-MUT**	F:CCAATAAATTATCATGCACGGAAAGTACTTGTGCGTTTG	62	533
	R:GCACAAGTACTTTCCGTGCATGATAATTTATTGG		
**ATF4-CDS**	F:CGGGATCCCGATGACCGAAATGAGCTTCCTG	60	1056
	R:CTCTAGAGCCTAGGGGACCCTTTTCTTCC		

Note: The underline letters the introduction of the restriction site of the enzyme.

### Cell transfection

Transfections were performed with Lipofectamine^TM^ 2000 using the liposome method. After the HUVECs reached 70%-80% confluency, they were transfected in 6-well plates. The cells were divided into 4 groups: the ATF4 plasmid group, the miR-1283 mimic group, the negative control group and the normal control group. First, the cells were washed with PBS. Then, they were resuspended in Opti-MEM^®^ I and plated in 6-well plates (5×10^5^ cells per well) at a final volume of 1 ml. Next, each group of cells was transfected. The final concentration of miRNA in each well was 0.1 mol/L [miR-1283 mimic group, 5 μl of miRNA dissolved in 245 μl of Opti-MEM^®^ I + 5 μl of Lipofectamine^TM^ 2000 dissolved in 245 μl of Opti-MEM^®^ I; negative control group, 5 μl of miRNA negative control solution in 245 μl of Opti-MEM^®^ I + 5 μl of Lipofectamine^TM^ 2000 dissolved in 245 μl of Opti-MEM^®^ I; normal control group, 500 μl of Opti-MEM^®^ I]. The cells were cultured at 37°C with 5% CO_2_ in a humidified incubator for 6 h. Then, the medium was replaced, and the cells were incubated for 48 h and collected.

### Animals

Thirty-two Kunming mice (22 ± 2 g) were purchased from the Experimental Animal Center of Guangdong Province (Guangzhou, China). The animals were housed with an alternate light-dark cycle (12 h temperature of 22 ± 2°C and a relative humidity of 50–60%). The mice were fed a complete formula, had access to water ad libitum, and underwent a one-week adaption period before the experiment.

The animal experiments were conducted in accordance with the Guide for the Care and Use of Laboratory Animals (2008, Washington, DC). The protocols for the animal studies were also reviewed and approved by the Experimental Animal Ethics Committee of Jinan University (Certification No: 20150624165336). The mice were randomly divided into a control group (n = 8), a miRNA-1283 antagomir (miRNA inhibitor) group (n = 8), a miRNA antagomir negative control group (n = 8), and a 4-PBA group (n = 8). The miRNA-1283 antagomir and 4-PBA groups received 80 mg/(kg•d) of the miRNA-1283 antagomir via tail vein injection on days 1–3; the control group received 80 mg/(kg•d) of physiological saline solution. After 3 days, all groups, except the 4-PBA group, which received 1 g/(kg•d) of 4-PBA, were given equal amounts of distilled water for 21 days (none of the mice died during the modeling process). The mice were monitored for daily intake of food, water and behavioral state. After the mice underwent 21 days of irrigation treatment, blood was obtained from the retro-orbital vein under inhaled ether anesthesia, and the samples were centrifuged to obtain serum. After the blood was collected, pressure on the site was sufficient to stop further bleeding. Finally, the mice were euthanized by cervical dislocation. The heart tissues were rapidly removed and washed with saline at 4°C, and part of the tissue was fixed with 4% paraformaldehyde. The remaining heart tissue was stored at –80°C for later use.

### Detection of NO, ET, EPCR, TM and vWF levels

Forty-eight hours after transfection, the supernatant was collected, and the OD values of nitric oxide (NO), ET, vWF, EPCR and TM in the supernatant were detected. Then, the expression levels of NO, ET, EPCR, TM and vWF were calculated according to the formulas described in the NO, ET, EPCR, TM and vWF kits.

Mouse serum was collected to determine the ET and NO content following the protocols provided with the mouse NO and ET kits.

### mRNA expression determined by qRT-PCR

Total RNA was extracted from the four groups. PCR was performed according to the manufacturer’s instructions, and the 2^-ΔΔCT^ method was used to determine the expression levels. Primers were designed based on the sequences in the NCBI gene database (Table 2).

**Table 2:** The sequences of the primers for qRT-PCR.

Gene	Sample	Primers	Annealing uniformity(°C)	Product Length (bp)
**ATF4**	human cell	F: 5'-CGCAACATGACCGAAATGAGCT-3'	58	132
		R: 5'-TTAGCCTTGTCGCTGGAGAACC-3'		
	mouse	F: 5'-GGTTCTGTCTTCCACTCCA-3'	59	81
		R: 5'-GGCTTCCTGTCTCCTTCA-3'		
**CHOP**	human cell	F: 5'-CTTGACCCTGCTTCTCTGGCTT-3'	58	155
		R: 5'-TTCCGTTTCCTGGTTCTCCCTT-3'		
	mouse	F: 5'-ACCTTCACTACTCTTGACCCT-3'	59	140
		R: 5'-TCTTCCTCCTCTTCCTCCT-3'		
**GRP78**	human cell	F: 5'-AAAGCTAAGAAGAAGGAACTGGAAG-3'	58	114
		R: 5'-CAACTCATCTTTTTCTGCTGTATCC-3'		
	mouse	F: 5'-GGCGTGAGGTAGAAAAGG-3'	59	151
		R: 5'-ATGGTAGAGCGGAACAGG-3'		
**PERK**	human cell	F: 5'-TACAAGAGGGAGAGGAACAAACGAA-3'	58	198
		R: 5'-CCTGTGAGGATGAGGATGGAAAAG-3'		
	mouse	F: 5'-GGGTGGAAACAAAGAAGAC-3'	59	96
		R: 5'-CAATCAGCAACGGAAACT-3'		
**GDPAH**	human cell	F: 5'-CATCAGCAATGCCTCCTGCAC-3	59	86
		R: 5'-TGAGTCCTTCCACGATACCAAAGTT-3'		
**BCL**_**2**_	mouse	F: 5'-ACAGAGGGGCTACGAGTG-3'	59	87
		R: 5'-GGCTGGAAGGAGAAGATG-3'		
**BAX**	mouse	F: 5'-GCTACAGGGTTTCATCCA-3'	59	170
		R: 5'-CGTCAGCAATCATCCTCT-3'		
**eIF 2a**	mouse	F: 5'-AACAAATGGAGAAAGTGCTG-3'	59	176
		R: 5'-ACCAGTCCCAAAGTCAAAC-3'		
**β-actin**	mouse	F: 5'-GGGAAATCGTGCGTGAC-3'	59	166
		R: 5'-AGGCTGGAAAAGAGCCT-3'		

### Protein detection

Proteins were extracted, and the protein concentration was determined using the BCA method. Total protein (30 μg) was added to a buffer sample for protein electrophoresis and was then transferred to a nitrocellulose membrane. The PVDF membrane was washed. Subsequently, the membrane was sealed and incubated with a diluted monoclonal antibody (1:1000) at 4°C overnight. Then, horseradish peroxidase IgG (1:12000) was added, and the membrane was incubated at 37°C for 1 h. After washing with TBST, diaminobenzidine was added for color development. We determined the quantitative values based on the absorbance of each band by Image J software.

### Hematoxylin-eosin(HE), Masson staining and TUNEL detection of apoptosis

Tissue fixation, dehydration, paraffin embedding, and slicing were conducted. After HE and Masson staining was performed, we followed the TUNEL kit protocol to detect apoptosis. The cells were observed under a light microscope. Photographs were obtained with a camera, and image analysis software was used to count the cells and perform the statistical analysis.

### Statistical analysis

All data were analyzed by one-way ANOVA and the distribution is presented as the mean ± standard error. A *P* value of less than 0.05 was considered statistically significant.

## Results

### Validation of the relationship between miR-1283 and ATF4

**Fig 2:**
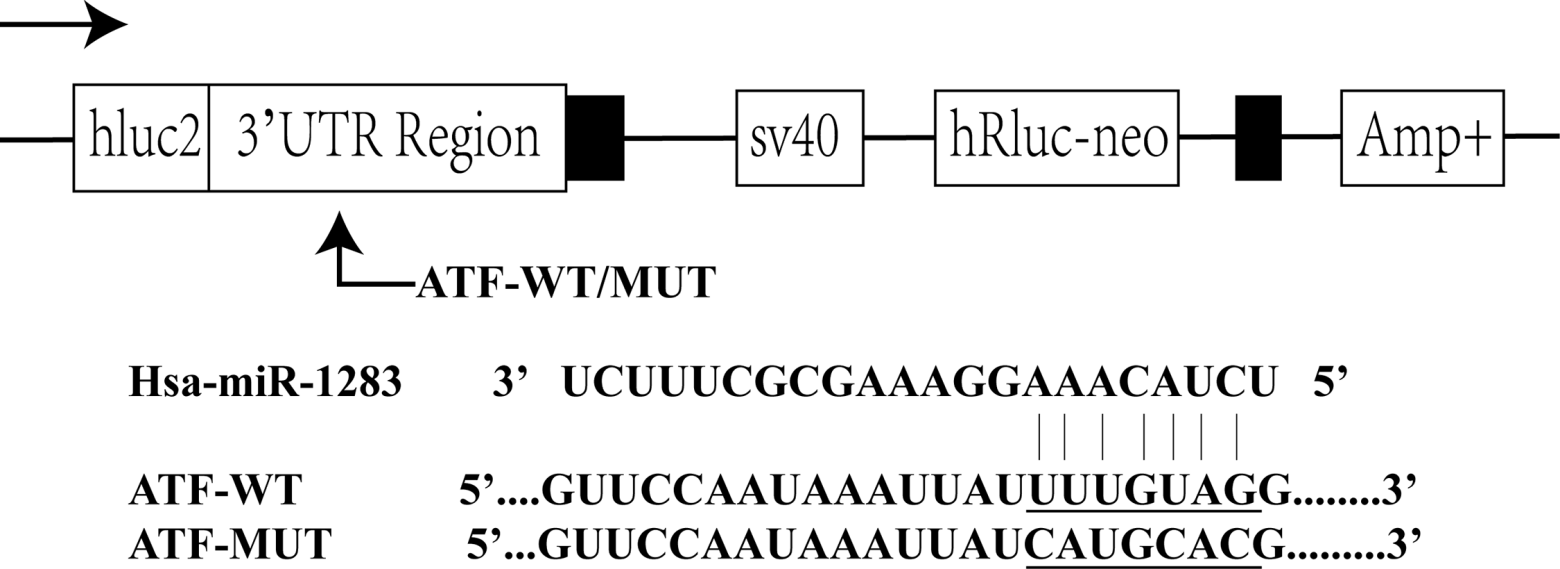
Dual-luciferase reporter assay. ATF4-WT indicates ATF4 wild-type luciferase.

**Fig 3:**
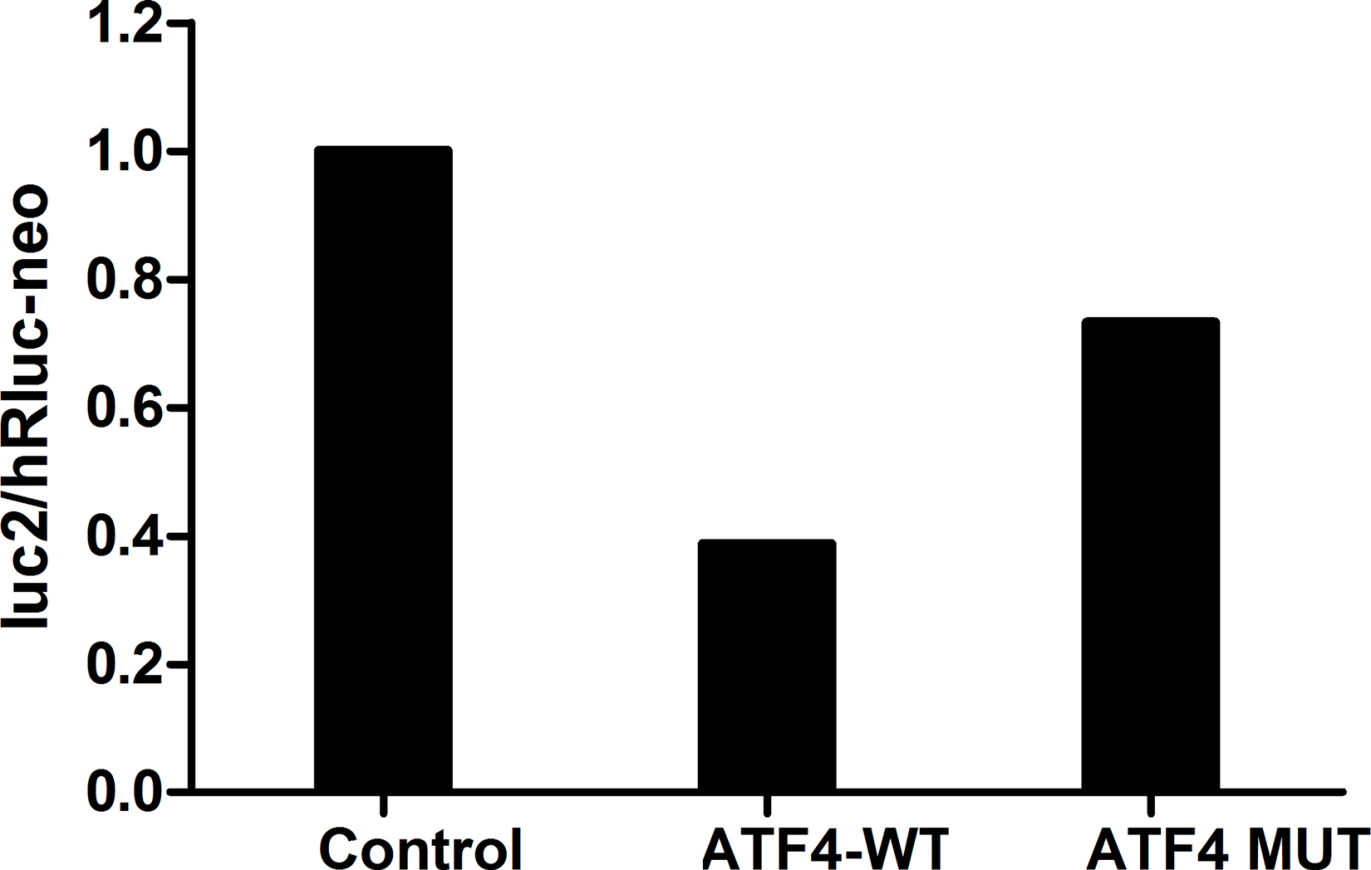
Validation of luc2/hRluc-neo between miR-1283 and ATF4.

The Dual-Luciferase Reporter Assay System was used to assess the relationship between ATF4 and miR-1283. Wild-type and mutant ATF4 were separately inserted at the 3′ end of the firefly luciferase gene (Fig 2). Then, the miR-1283 mimic was co-transfected with a no-insert control or an ATF4 wild-type or mutant luciferase reporter into the HUVECs. Compared to the no-insert control (Control), the firefly luciferase activity of the wild-type ATF4 luciferase reporter (ATF4-WT) was 38.70%, but it was 73.20% with the mutant ATF4 luciferase reporter (ATF4-Mut) (Fig 3). These results showed that miR-1283 significantly decreased the firefly luciferase activity of the wild-type ATF4 reporter.

### miR-1283 and ATF4 affect the expression of PERK-eIF2α-ATF4 in vascular endothelial cells

**Fig 4:**
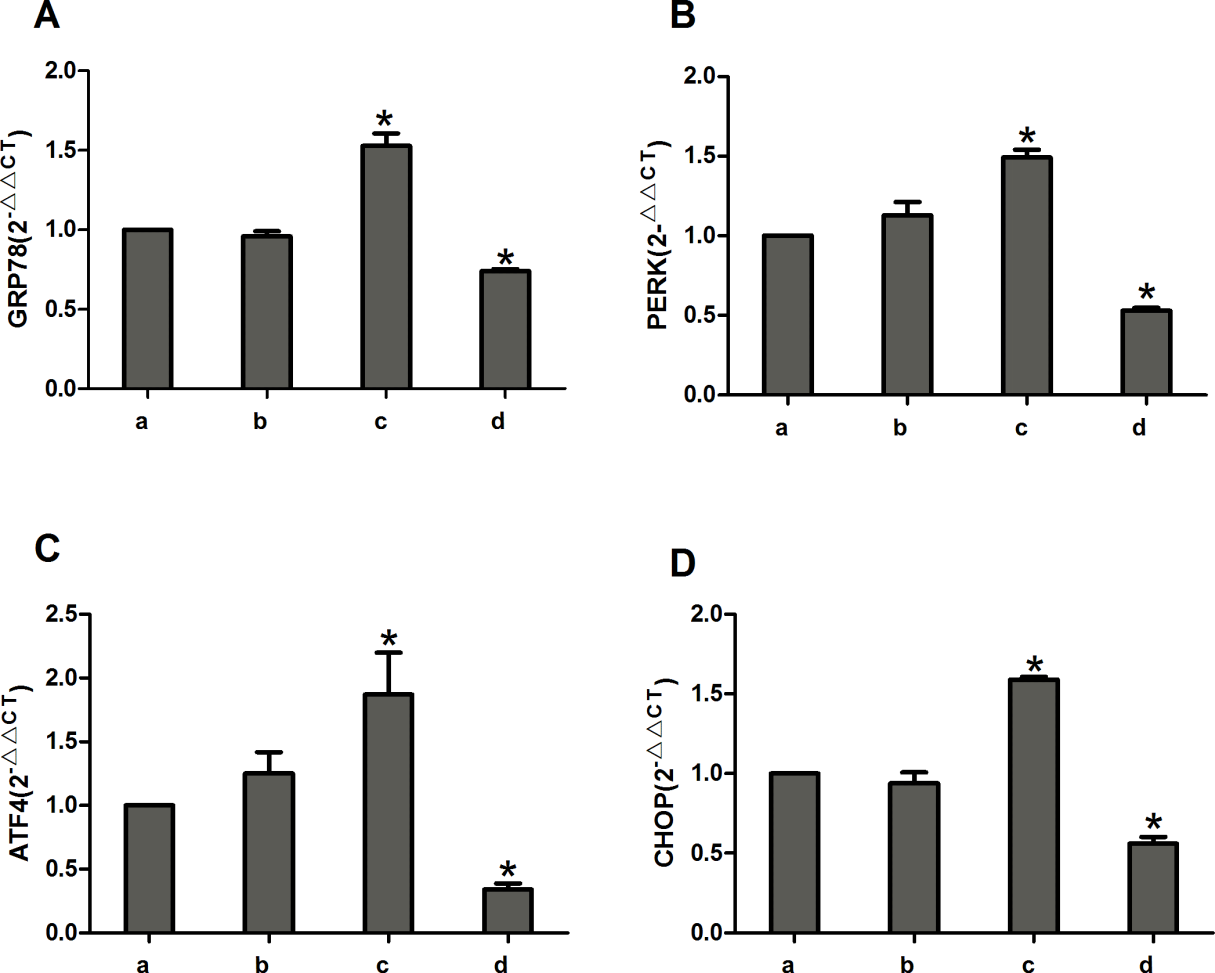
The expression of GRP78, PERK, ATF4 and CHOP mRNA in the transfected cells. **(A) The mRNA expression of GRP78; (B) The mRNA expression of PERK; (C) The mRNA expression of ATF4; (D) The mRNA expression of CHOP.** Note: A. normal group, B. negative group, C. ATF4 overexpress group, and D. miR-1283 mimic group.* *P*<0.05 (compared with a. normal control group).

Compared to the control group, the miR-1283 mimic group had decreased mRNA levels of GRP78, PERK, ATF4 and CHOP (*P*<0.05). In contrast, the levels were significantly increased in the ATF4 overexpression group (*P*<0.05), and the differences were not significant between the normal and the negative control groups (*P*>0.05) (Fig 4).

**Fig 5:**
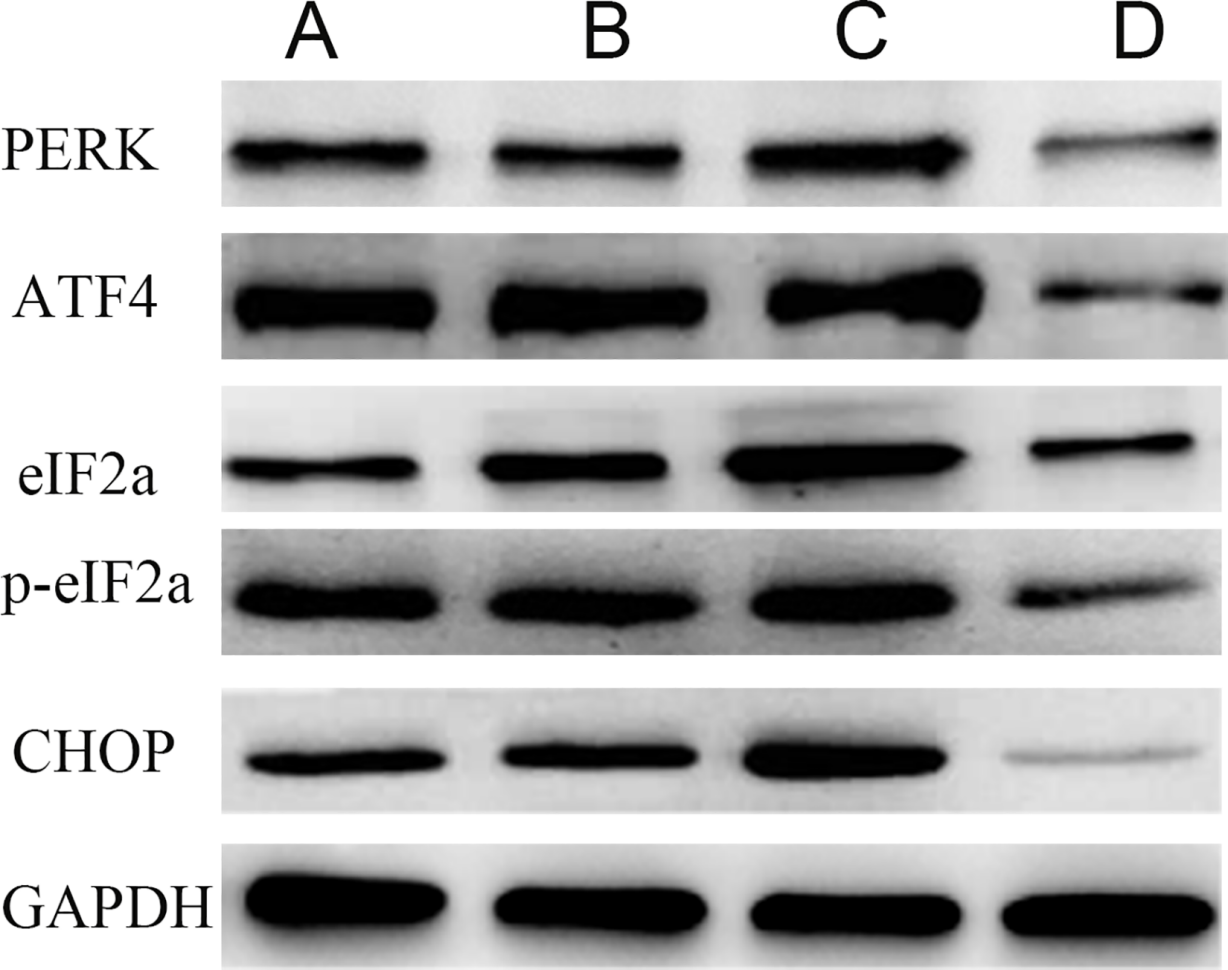
The picture of PERK, ATF4, eIF2a, p- eIF2a and CHOP proteins in the transfected cells. Note: A. normal group, B. negative group, C. ATF4 overexpress group, and D. miR-1283 mimic group.

**Fig 6:**
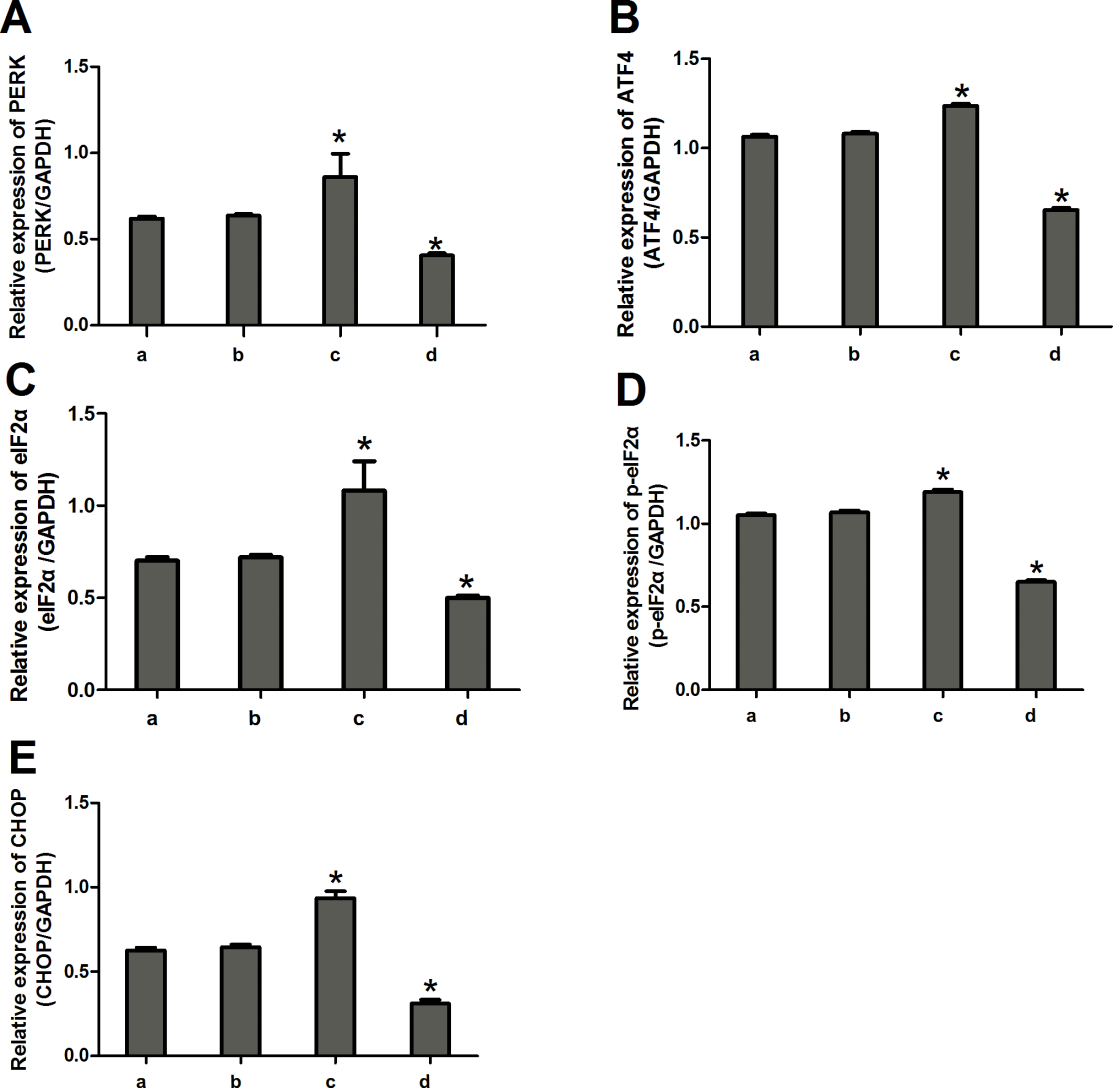
The expression of PERK, ATF4, eIF2a, p-eIF2a and CHOP proteins in the transfected cells. **(A) The protein expression of PERK; (B) The protein expression of ATF4; (C) The protein expression of eIF2a; (D) The protein expression of p-eIF2a; (E) The protein expression of CHOP.** Note: a. normal group, b. negative group, c. ATF4 overexpress group, d. miR-1283 mimic group. * *P*<0.05 (compared with a. normal group).

Compared to the control group and the miRNA antagomir negative control group, the miR-1283 mimic group had decreased protein levels of PERK, ATF4, eIF2α, p-eIF2α and CHOP (*P*<0.05). The levels of these proteins were significantly increased in the ATF4 overexpression group (*P*<0.05), and the differences were not significant between the normal and the negative control groups (Figs 5 and 6).

### miR-1283 and ATF4 affect vascular endothelial cell injury

The HUVECs were transfected with the miR-1283 mimic or pcDNA3.1 with ATF4-CDS, and the expression of several factors in the culture medium and the cells was measured after 48 h.

**Fig 7:**
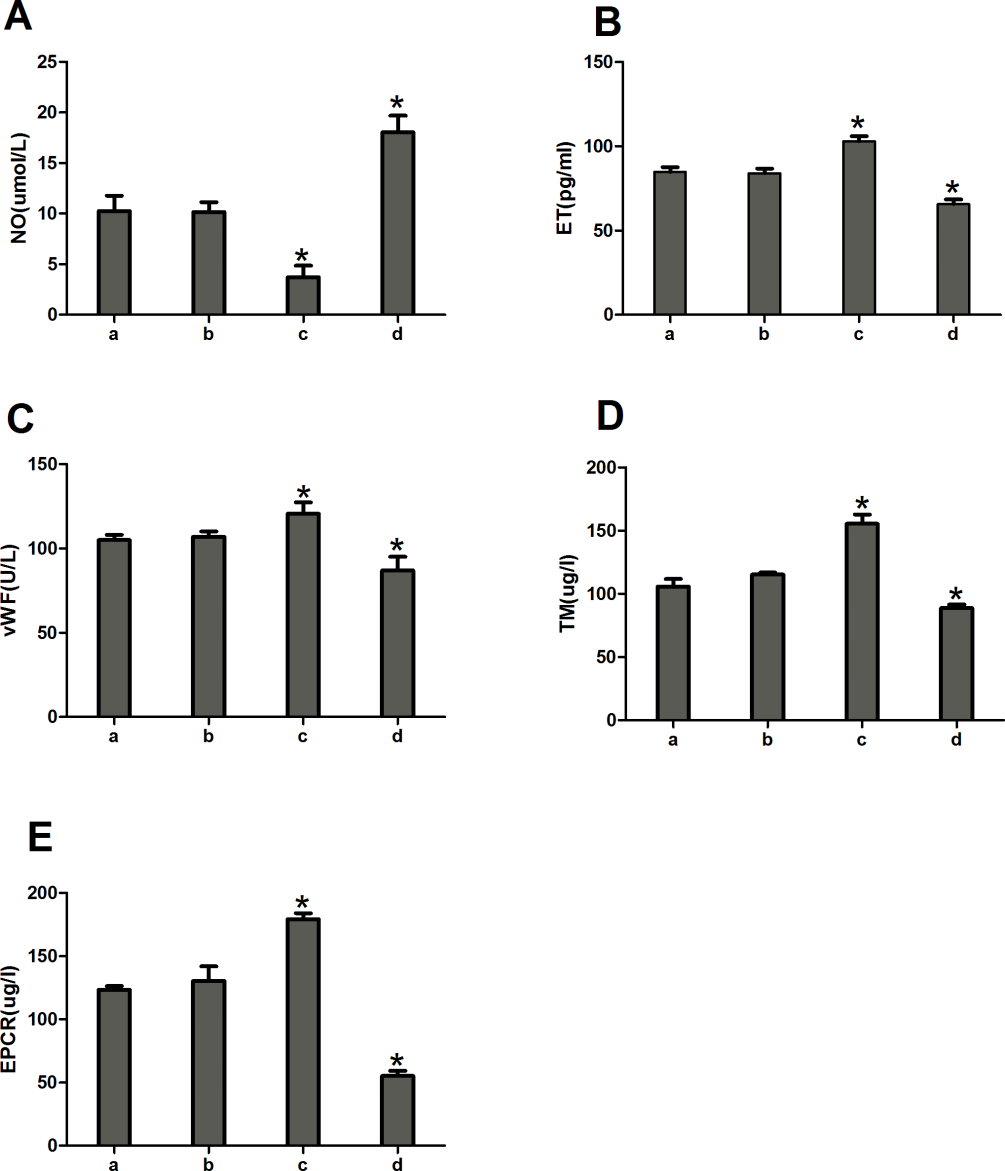
The expression of NO, ET, vWF, TM and EPCR in the transfected cells. **(A) The expression of NO; (B) The expression of ET; (C) The expression of vWF; (D) The expression of TM; (E) The expression of EPCR.** Note: a. normal control group, b. miRNA mimic negative group, c. ATF4 overexpress group, d. miR-1283 mimic group. * *P*<0.05 (compared with a. normal control group).

Compared to the normal and negative control groups, the miR-1283 mimic group showed significantly increased NO levels, and the levels of ET, vWF, TM, and EPCR were significantly decreased (*P*<0.05). In contrast, the level of NO was decreased, and the levels of ET, vWF, TM, EPCR were increased in the ATF4 overexpression group (*P*<0.05). The differences were not significant between the normal and the negative control groups (*P*>0.05) (Fig 7).

### The expression of PERK-eIF2ɑ-ATF4 in mouse heart tissues

**Fig 8:**
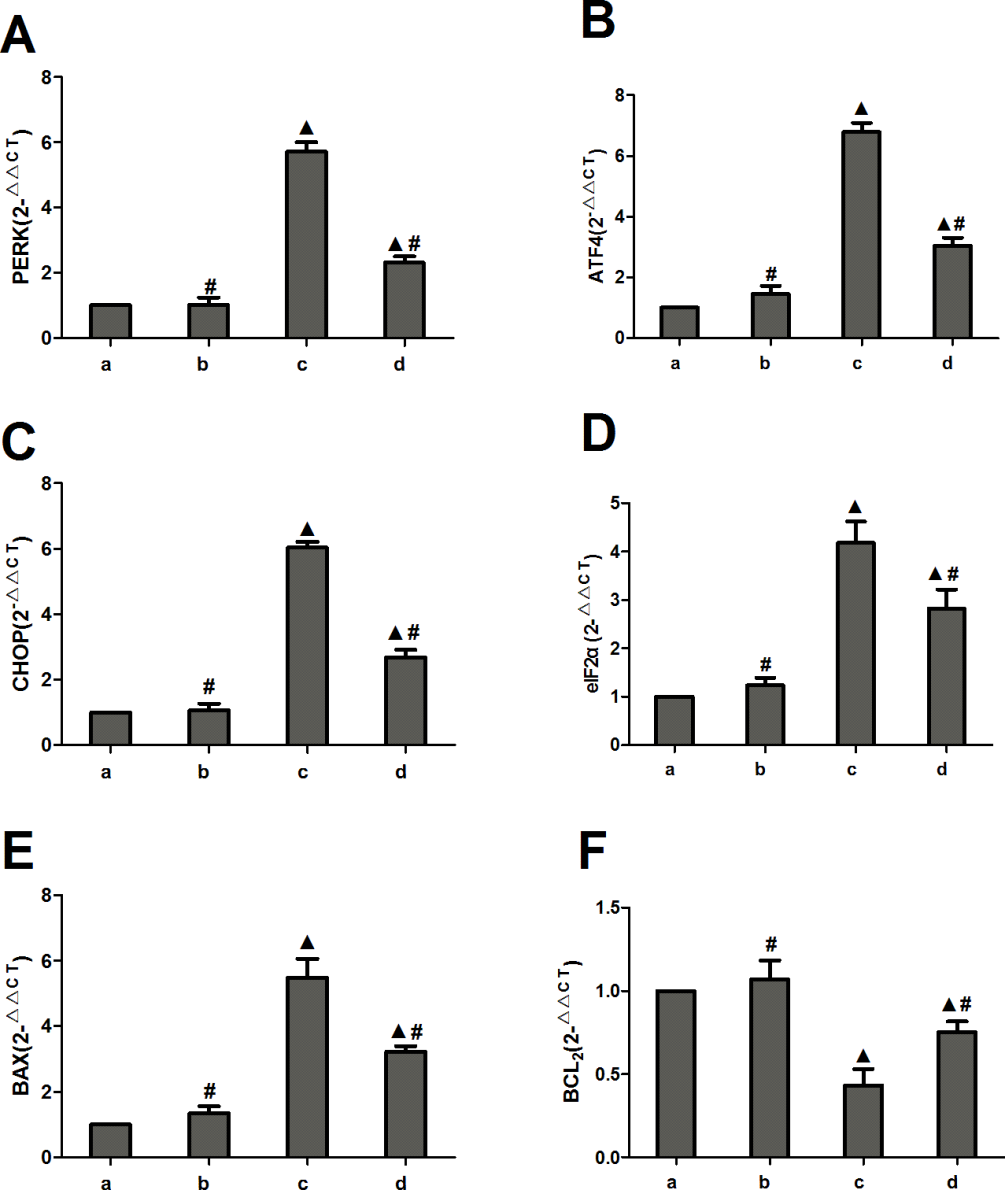
The expression of PERK, ATF4, eIF2a, CHOP, Bax and Bcl2 mRNA in mouse heart tissue. **(A) The mRNA expression of PERK; (B) The mRNA expression of ATF4; (C) The mRNA expression of CHOP; (D) The mRNA expression of eIF2a; (E) The mRNA expression of BAX; (F) The mRNA expression of BCL2**. Note: a. normal group, b. miRNA antagomir negative group, c. miR-1283 antagomir group, d. 4-PBA group.^▲^, *P*<0.05 (compared with a. normal control group); ^**#**^, *P*<0.05 (compared with c. miR-1283 antagomir group).

Compared to the normal and miRNA antagomir negative control groups, the miR-1283 antagomir group had increased PERK, ATF4, CHOP, eIF2α, and Bax mRNA levels, while BCL2 was decreased (*P*<0.05). Compared to the miR-1283 antagomir group, the 4-PBA group had decreased PERK, ATF4, CHOP, eIF2α, and Bax mRNA levels, and the BCL2 level was increased (*P*<0.05). No significant differences were observed between the normal and miRNA antagomir negative control groups (*P*>0.05) (Fig 8).

**Fig 9:**
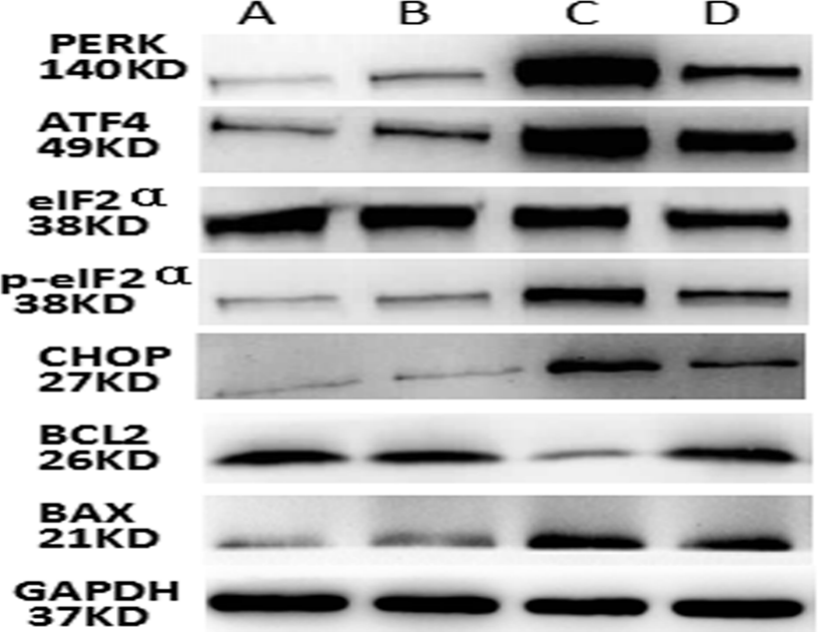
The picture of PERK, ATF4, eIF2a, p-eIF2a CHOP, Bax and Bcl2 proteins in mouse heart tissue. Note: A. normal group, B. miRNA antagomir negative group, C. miRNA-1283 antagomir group, D. 4-PBA group.

**Fig 10:**
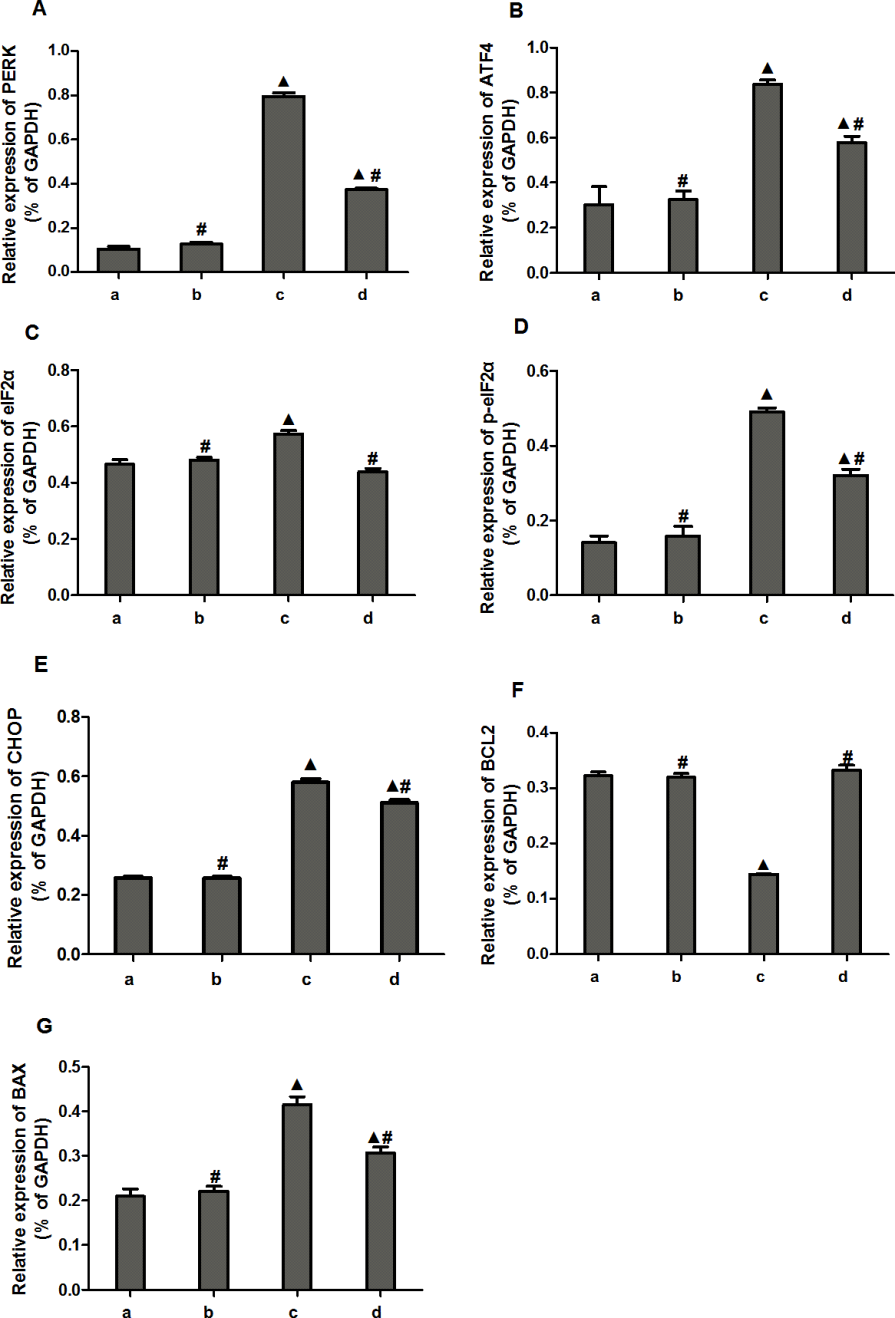
The expression of PERK, ATF4, eIF2a, p- eIF2a, CHOP, Bax and BCL2 proteins in mouse heart tissue. **(A) The protein expression of PERK; (B) The protein expression of ATF4; (C) The protein expression of eIF2a; (D) The protein expression of p-eIF2a; (E) The protein expression of CHOP; (F) The protein expression of BCL2; (G) The protein expression of Bax.** Note: a. normal group, b. miRNA antagomir negative group, c. miR-1283 antagomir group, d. 4-PBA group. *, *P*<0.05 (compared with a. normal control group); ^**#**^, *P*<0.05 (compared with c. miR-1283 antagomir group).

Compared to the normal and miRNA antagomir negative control groups, the miR-1283 antagomir group showed increased protein expression levels of PERK, ATF4, p-eIF2α, eIF2α, BAX and CHOP, while the BCL2 level was decreased (*P*<0.05). Compared to the miR-1283 antagomir group, the 4-PBA group showed decreased protein levels of PERK, ATF4, p-eIF2α, eIF2α, BAX and CHOP, while the BCL2 level was increased (*P*<0.05). However, no significant differences were observed between the normal and miRNA antagomir negative control groups (*P*>0.05) (Figs 9 and 10).

### Influence of the miR-1283 antagomir on vascular endothelial injury in mice

**Fig 11:**
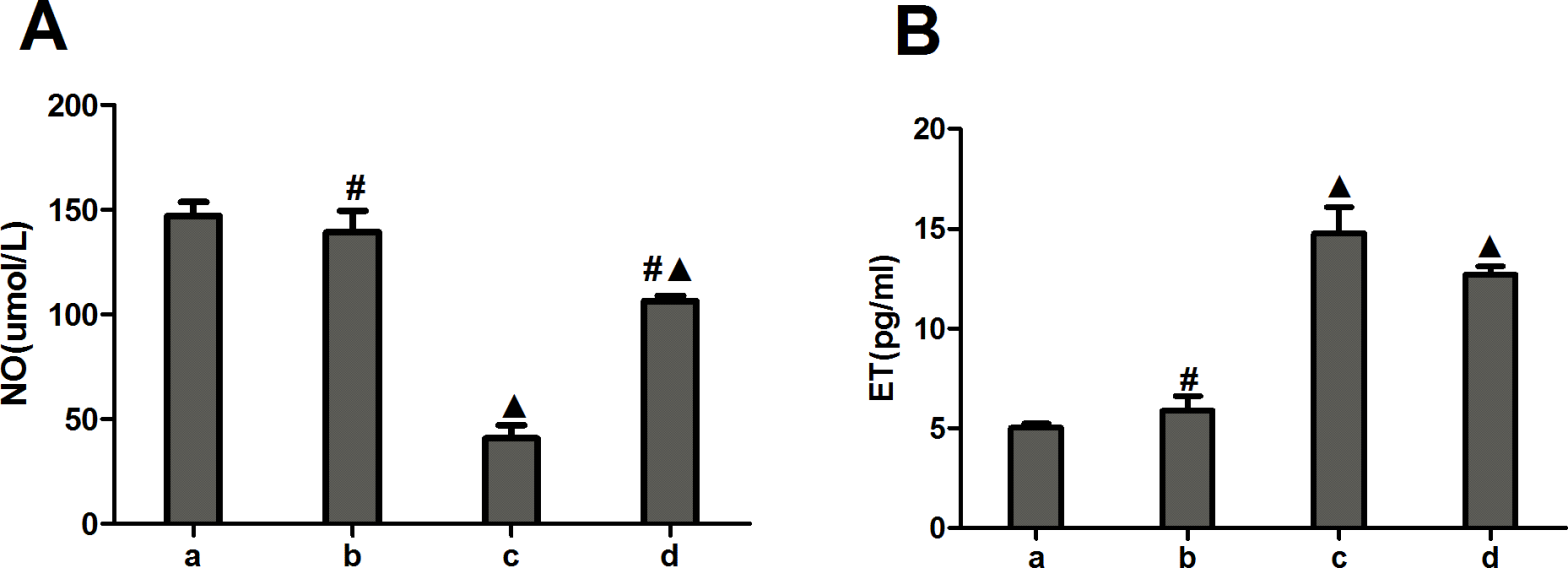
The expression of NO and ET in mouse serum. **(A) The expression of NO**; **(B) The expression of ET.** Note: a. normal group, b. miRNA antagomir negative group, c. miR-1283 antagomir group, d. 4-PBA group. ^▲^, *P*<0.05 (compared with a. normal control group); ^**#**^, *P*<0.05 (compared with c. miR-1283 antagomir group).

The NO level was significantly lower (*P*<0.05) and the ET level was significantly higher (*P*<0.05) in the miR-1283 antagomir group than in the normal control group. The NO level was significantly higher (*P*<0.05) and the ET level was significantly lower (*P*<0.05) in the 4-PBA group than in the miR-1283 antagomir group. No significant difference was observed between the normal group and the miRNA antagomir negative control group (*P*> 0.05) (Fig 11).

### miR-1283 antagomir damage to the heart tissues of mice

**Fig 12:**
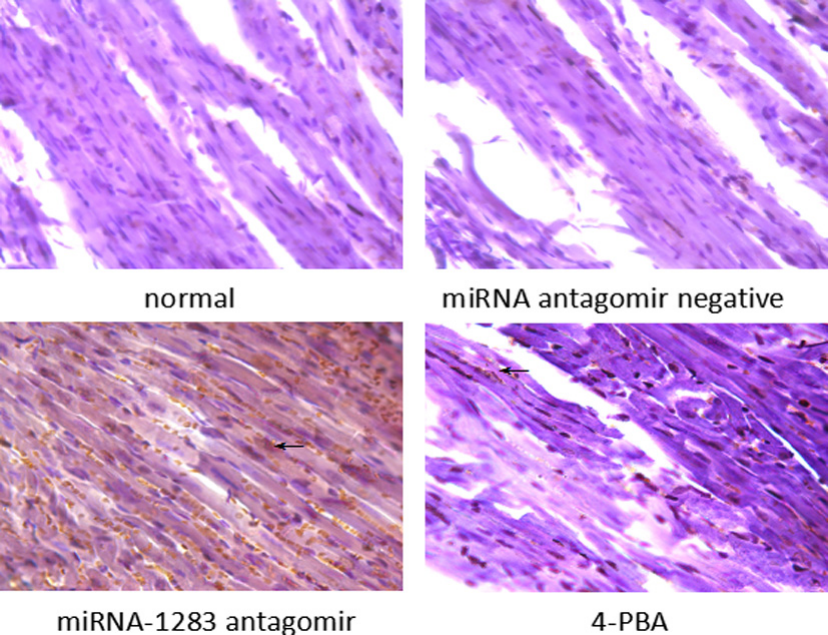
The picture of TUNEL cells apoptosis in mouse heart tissue.

**Fig 13:**
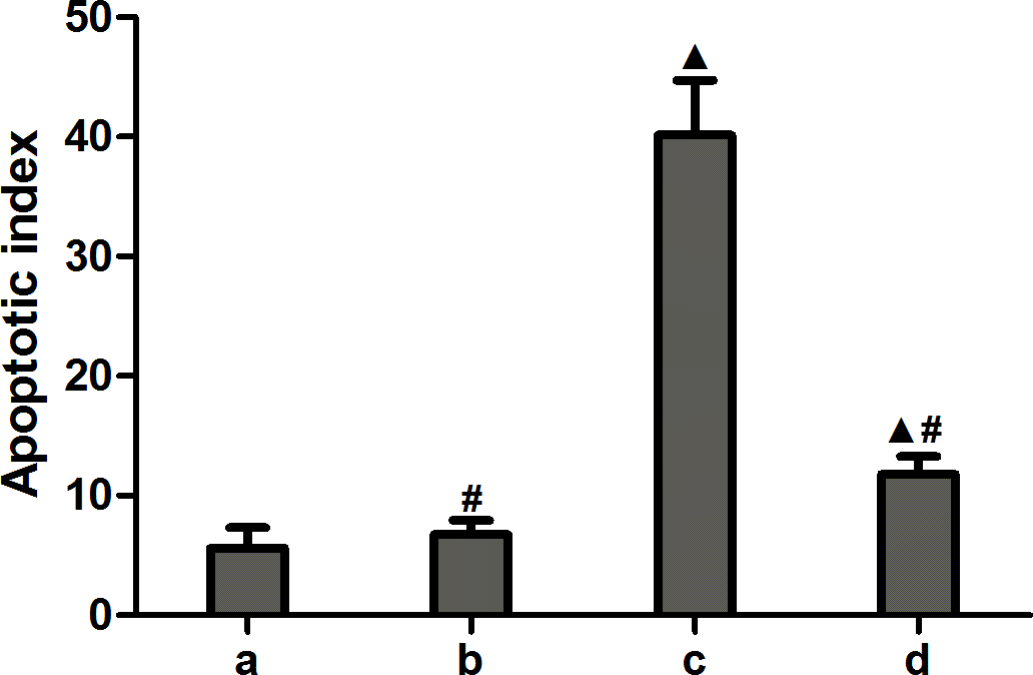
The expression of TUNEL cells apoptosis in mouse heart tissue. Note: a. normal group, b. miRNA antagomir negative group, c. miR-1283 antagomir group, d. 4-PBA group. ^▲^, *P*<0.05 (compared with a. normal control group); ^**#**^, *P*<0.05 (compared with c. miR-1283 antagomir group).

TUNEL-positive staining was indicated by brown particles located in the nuclei of the heart cells. Apoptosis of heart tissues in the normal and miRNA antagomir negative control groups was not obvious. The miRNA-1283 antagomir group showed extensive TUNEL-positive staining. TUNEL staining was decreased in the 4-PBA group, indicating that apoptosis was reduced (Figs 12 and 13).

**Fig 14:**
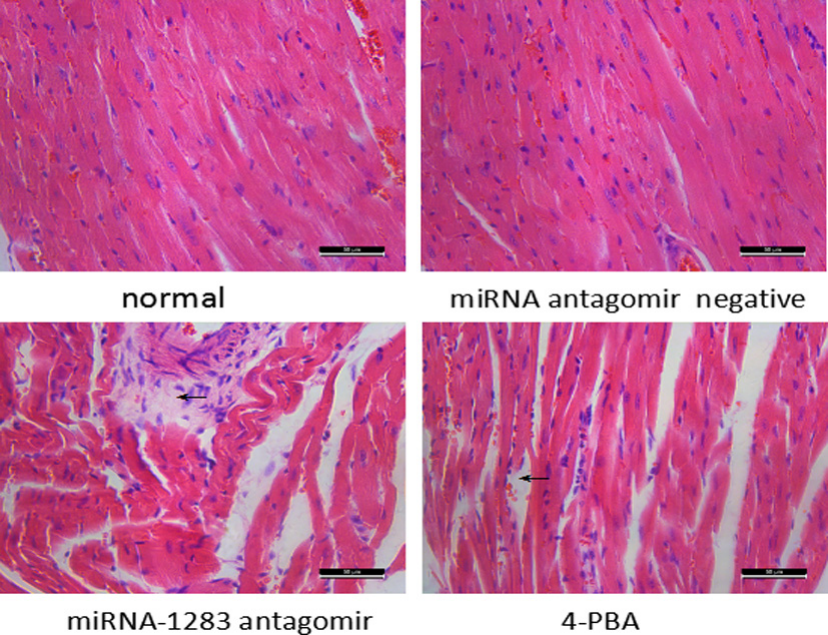
The picture of HE staining in mouse heart tissue.

HE staining of myocardial pathological sections showed that samples from the normal control group and the miRNA antagomir negative control group exhibited an ordered, clear cellular structure. The lesions in the miRNA-1283 antagomir group samples showed disordered myocardial cell arrangement, swelling, degeneration, and necrosis accompanied by inflammatory cell infiltration. The lesions were ameliorated in the 4-PBA group compared to the miR-1283 antagomir group (Fig 14).

**Fig 15:**
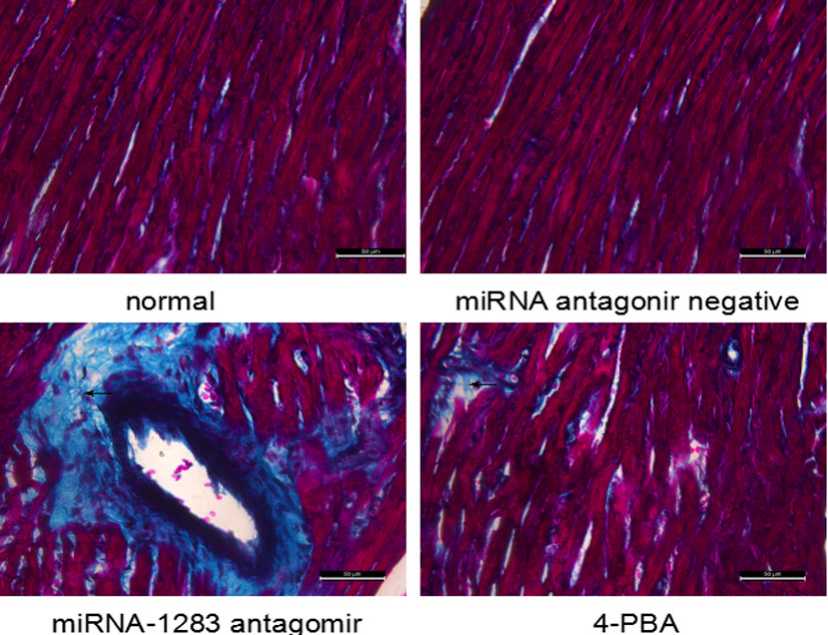
The picture of Masson staining in mouse heart tissue.

The collagen fibers appeared green, the myocardial fibers were red, and red blood cells were orange following Masson staining. The normal and miRNA negative control groups exhibited red muscle fibers, and few green collagen fibers were observed in the myocardial cells and blood vessels. In the areas with myocardial interstitial collagen, which was increased in mouse tissues in the miRNA-1283 antagomir group, the myocardial cell structure was disordered, the nucleus or cytoplasm exhibited vacuoles and degeneration, damaged myocardium was replaced by collagen tissue, and inflammatory cell infiltration could be observed. Part of this region was replaced by collagen tissue. The lesions were ameliorated in the 4-PBA group compared to the miR-1283 antagomir group (Fig 15).

## Discussion

miRNAs, a type of non-protein-coding RNA molecule, play an important role in regulating gene expression. Recent studies have shown that miRNAs are involved in the formation and regulation of ERS [6–8], and interactions between ERS and miRNAs are involved in the development of diseases. ATF4 is a key molecule that increases in ERS-induced apoptosis [9]. Inhibition of miR-1283 in HA-VSMCs increased the expression of ATF1 mRNA as well as the ROS levels. Additionally, it may be involved in essential hypertension [10]. In our previous study, we found that ATF4 is highly expressed in damaged vascular endothelial cells and may be a target of miR-1283 [5]. In this study, we confirmed this hypothesis using dual-luciferase reporter assays (Fig 3). The results showed that overexpression of miR-1283 downregulated ATF4 mRNA (Fig 4C) and protein expression (Figs 5 and 6) using qPCR and western blot analyses. These results indicated that ATF4 is a target gene of miR-1283.

The ER is an important subcellular organelle that is involved in the post-translational modification and folding of cellular proteins. ATF4 is an activating transcription factor and also a key molecule in induction of apoptosis during ERS [11]. It plays an important role in the PERK-eIF2α-ATF4-CHOP pathway, which is one of the ERS signaling pathways. When ERS occurs, PERK and GRP78 dissociate, and activated PERK promotes the phosphorylation of elF2a. p-eIF2α upregulates the expression of ATF4 and several downstream genes and enhances the activation of CHOP, which induces apoptosis in cells [12]. In this study, we also found that ATF4 overexpression upregulated the mRNA and protein expression of the downstream gene CHOP in HUVECs and mice, and ATF4 additionally upregulated the expression of the upstream genes PERK, eIF2α and p-eIF2α (Figs 4–6). The overexpression of ATF4 increased activation of the PERK-eIF2α-ATF4 pathway, which may promote ERS. That may lead to activation of the genes upstream of ATF4 in the PERK-eIF2α-ATF4 pathway.

Nevertheless, the results suggested that inhibition of miR-1283 promoted ERS by targeting ATF4. At the same time, ERS stimulates apoptosis. This was also confirmed in our study. A miR-1283 inhibitor increased apoptosis in heart tissues as determined by TUNEL analysis (Figs 12 and 13). Additionally, the expression of the apoptosis-related genes BAX and GPR78 was also elevated. Several reports have shown that ERS-NO, the misfolded protein response and inflammation are linked in the pathogenesis of many inflammatory diseases [13–14]. Moreover, ERS is not only involved in the inflammatory response but is also an important cause of inflammation [15–17]. In this study, we also found that inhibition of miR-1283 increased cardiovascular inflammation as determined by HE and Masson staining. Recently, a report showed that ERS coupled with inflammatory activation induced vascular endothelial cell injury by promoting the expression of p38 MAPK and its downstream molecules and oxidative stress [18]. The results of this study also showed that inhibition of miR-1283 or ATF4 overexpression promoted vascular endothelial cell injury. The level of NO (an in vivo signaling molecule and a protective factor for VECs) decreased, and the levels of ET (which damages VECs by strongly contracting vascular smooth muscles), vWF (a marker of vascular injury or vascular functional disturbances [19–20]), TM (not only an important anticoagulant cofactor but also a molecular marker of VEC damage) and EPCR (has a role in anticoagulation by inhibiting factor V and VIII [21–22]) significantly increased (Figs 7 and 11).

In summary, inhibition of miR-1283 promoted ERS by regulating the PERK/ATF4 pathway via inhibition of ATF4, which stimulated apoptosis and inflammation and eventually led to vascular endothelial injury.

## Supporting Information

S1 File Fund Certificates. (RAR)
